# Comparing the Accuracy of Markerless Motion Analysis and Optoelectronic System for Measuring Gait Kinematics of Lower Limb

**DOI:** 10.3390/bioengineering12040424

**Published:** 2025-04-16

**Authors:** Luca Emanuele Molteni, Giuseppe Andreoni

**Affiliations:** 1 Scientific Institute IRCCS “E. Medea”, Bosisio Parini, 23842 Lecco, Italy; 2 Department of Design, Politecnico di Milano, 20133 Milano, Italy

**Keywords:** kinematic, markerless, Openpose, pediatric

## Abstract

(1) Background: Marker-based optical motion tracking is the gold standard in gait analysis; however, markerless solutions are rapidly emerging today. Algorithms like Openpose can track human movement from a video. Few studies have assessed the validity of this method. This study aimed to assess the reliability of Openpose in measuring the kinematics and spatiotemporal gait parameters. (2) Methods: This analysis used simultaneously recorded video and optoelectronic motion capture data. We assessed 20 subjects with different gait impairments (healthy, right hemiplegia, left hemiplegia, paraparesis). The two methods were compared using computing absolute errors (AEs), intraclass correlation coefficients (ICCs), and cross-correlation coefficients (CCs) for normalized gait cycle joint angles. (3) Results: The spatiotemporal parameters showed an ICC between good to excellent, and the absolute error was very small: cadence AE = 1.63 step/min, Mean Velocity AE = 0.16 m/s. The Range of Motion (ROM) showed a good to excellent agreement in the sagittal plane. Furthermore, the normalized gait cycle CCC values indicated moderate to strong coupling in the sagittal plane. (4) Conclusions: We found Openpose to be accurate for sagittal plane gait kinematics and for spatiotemporal gait parameters in the healthy and pathological subjects assessed.

## 1. Introduction

The measurement of human motion represents one of the most interesting and challenging topics of metrology. Moreover, measuring and analyzing movement is important for many fields, including medicine, sports, and physical rehabilitation. In particular, in the context of physical rehabilitation, where over 1 billion individuals, approximately 15% of the global population, live with some form of disability [1]. In this context, reliable motion capture is essential, as it enables quantitative assessment of treatment progress and eliminates the reliance on subjective visual evaluation, which is susceptible to evaluator bias and poor inter-rater reliability. Furthermore, integrating motion capture technology into the treatment of physical impairments has the potential to significantly enhance therapeutic outcomes [2,3]. Currently, marker-based optical motion tracking systems offer the highest metrological performance, particularly in terms of marker localization accuracy (typically on the order of tenths of a millimeter), as well as measurement repeatability and sampling frequency [4]. In these systems, joint positions and body segment orientations are derived from the three-dimensional localization of passive (or occasionally active) markers, which are attached to the subject’s body and tracked using a calibrated multi-camera stereophotogrammetric system [5]. These technologies are very useful but present some limits; indeed, they are expensive, complex, and require trained personnel to conduct the recording and place the markers on specific anatomical landmarks. Marker-based systems, therefore, are mainly used in specialized laboratories for clinical/rehabilitation applications [5,6,7].

In the last decades, thanks to the growth of remote telemonitoring, the interest in markerless solutions has rapidly grown for applications of motion analysis, in particular with the aim to reduce the cost and to simplify the process [8,9,10]. Markerless systems rely on four key components: a camera system, a body model, the extracted image features, and the algorithms responsible for estimating the model’s shape, pose, and position [8]. In these approaches, two families of camera systems can be used: the “depth map” camera and the “traditional” camera. The first family, the “depth map” camera, produces an image where each pixel describes the distance of a point in the scene from the camera. For example, in this family, we can find Microsoft Kinect (Microsoft Corporation, Redmond, WA). These solutions are particularly effective for real-time full-body pose estimation in interactive systems and video games [11,12,13]; however, they also have limitations that hinder their wide application in clinical or biomechanical settings, namely, a short range, non-usability in bright sunlight, and potential interference between multiple sensors [8]. The second family, the “traditional” camera, produces a “traditional” image that can be elaborated with the novel artificial intelligence algorithms based on automatic landmark identification on video images (computer vision). This method has paved the way for a new approach to markerless motion capture, potentially implementable using low-cost hardware [8,10,14]. In this method, machine learning techniques were exploited to identify the nodes of a skeletal structure describing the posture of a human subject within a given image frame [15]. Joint detection of the human body, hand, and foot is now possible with various systems, such as Openpose [16,17,18]. Openpose takes as input color images from simple web-cameras and, using a two-branch convolutional neural network (CNN), produces as output confidence maps of key points and affinity for each key point pair (that is, belonging to the same skeleton). Although promising results have been achieved, the development of markerless systems capable of reliably reconstructing human motion in a timely, unobtrusive, and valid manner remains an open challenge [8]. The growth of studies on the application of these methods is rapid. However, only a few studies have evaluated Openpose’s performance in computing lower limb angles during gait using videos acquired with a single camera and comparing them to multi-camera marker-based systems [19,20,21,22,23,24]. This is particularly important considering that abnormal gait kinematics and spatiotemporal parameters can occur after injuries and often coincide with disability [25,26]. Hence, knowledge of software packages for position estimation that are accurate to measure the kinematics of the step can help in treating individuals with neurological lesions, orthopedic lesions, and amputees.

This study aimed to compare these pre-trained algorithms in terms of reliability for gait kinematics and spatiotemporal gait parameters. In particular, using gait videos acquired in the sagittal and frontal planes without requiring preliminary operations such as calibration. In order to assess these parameters in a simple, fast, and inexpensive way and facilitate easy clinical application, we sought to assess the reliability of Openpose in a sample of pediatric subjects, with and without impairments, during gait.

## 2. Materials and Methods

### 2.1. Subjects

In this study, we employed an existing dataset that consists of simultaneous video and motion capture recordings. From this dataset, we extracted twenty subjects. Specifically, we selected a heterogeneous group of subjects with different types of gait impairments to assess whether Openpose could reliably measure lower limb joint angles even in the presence of altered gait patterns. The subjects, aged between 5 and 18 years, had varying pathological histories and motor impairments and were divided into four groups based on the following inclusion criteria:

- Five healthy subjects without motor disabilities, with no history of serious injuries (e.g., ligament or musculoskeletal injuries, neurological disorders, fractures) and no history of surgical procedures on the extremities or trunk (4 males/1 females; mean age: 9.24–SD 3.98; age range: 5.4–15.0).
- Five subjects with a diagnosis of cerebral palsy (CP) and right hemiplegia, able to walk more than 30 m without assistance, orthotics, or aids (3 males/2 females; mean age: 11.44–SD 3.55; age range: 7.6–15.9).
- Five subjects with a diagnosis of cerebral palsy and left hemiplegia, able to walk more than 30 m without assistance, orthotics, or aids (2 males/3 females; mean age: 10.88–SD 3.16; age range: 6.8–14.5).
- Five subjects with a diagnosis of spastic paraparesis, able to walk more than 30 m without assistance, orthotics, or aids (3 males/2 females; mean age: 12.44–SD 3.46; age range: 9.4–17.7).

A summary of the sample’s baseline characteristics is provided in Table 1.

The study was carried out in accordance with the 1964 Helsinki Declaration and its later amendments. The Ethics Committee “Comitato Etico Lombardia 2” approved the study (Study ID: 1121/Andreoni, date of approval 15 May 2024).

### 2.2. Data Collection

All measurements were collected using an optoelectronic multi-camera system for human motion analysis (SMART DX, BTS SpA, Milan, Italy), consisting of eight high-resolution infrared cameras operating at a sampling frequency of 100 Hz, which tracked the position of passive retroreflective markers placed directly on the subject’s skin. In accordance with the Davis Protocol, 22 markers (plastic spheres covered with reflective film, 10 mm in diameter) were placed for the experimental procedure [27]. Marker placement was performed by a physiotherapist specifically trained in the use of optoelectronic systems for human motion analysis. During the data acquisition protocol, the subject was asked to walk barefoot on a 6 m distance at a self-selected normal-pace speed.

**Figure 1:**
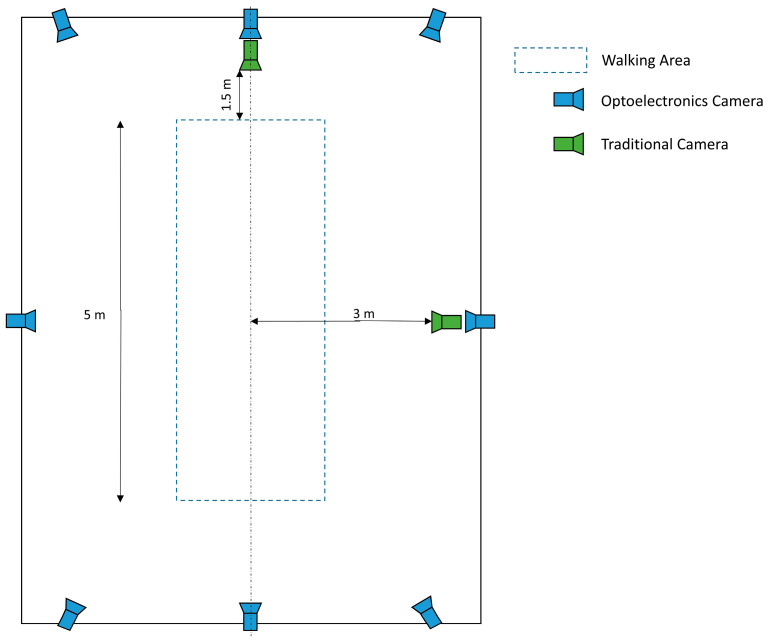
Gait lab scheme.

Video data were simultaneously recorded with 2 cameras (eVIXTA frequency: 25 fps; resolution: 640 × 480). As in previous studies with similar objectives [1,20,23], the two cameras were mounted on tripods at a height of approximately 90 cm. The first camera was placed about 3 m from the center of the walkway, aligned perpendicularly to the subject’s sagittal plane. The second camera was positioned at 1.5 m from the start/end of the walk area, perpendicular to the subject’s frontal plane. During the trial, the subject walked indifferently from the right to the left of the room or vice versa at a self-selected normal pace (Figure 1).

### 2.3. Data Processing

The motion capture system’s raw data were processed using Smart Analyzer software 1.10.0470 (BTS Bioengineering, Milano, Italy). First, the 3D data were filtered and interpolated using spline cubic interpolation in the case of missing data for a short time. Subsequently, spatiotemporal parameters (including cycle duration, cadence, gait speed, stance phase, swing phase, double-support phase, stride length, and step width) were computed, along with conventional kinematic parameters based on the standard Davis marker-set protocol [27].

**Figure 2:**
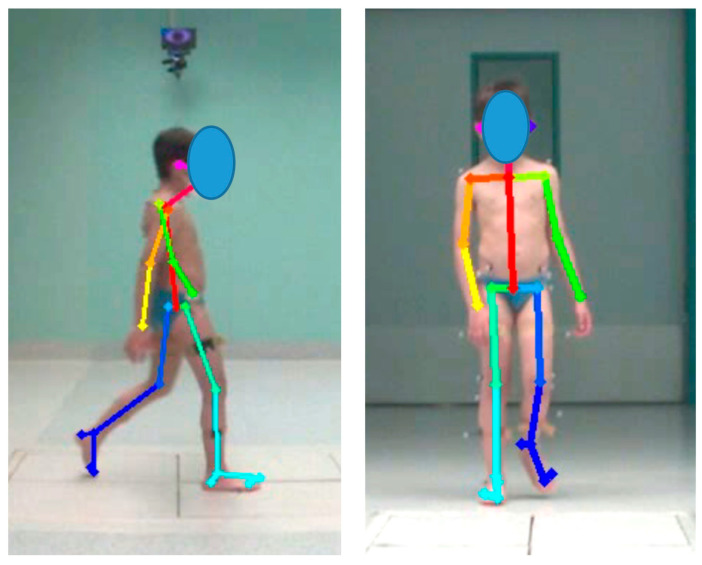
Location of body landmarks on sagittal plane and frontal plane.

The two videos were processed using Openpose, which returns a set of 25 2D key point coordinates for body pose estimation (in pixels) for each video. Key points were located at relevant body landmarks (e.g., left foot, right foot, face) and were used to determine 2D Cartesian coordinates in the sagittal and frontal planes (Figure 2).

Using Matlab R2024a, the data that were calculated using routines were filtered and interpolated with linear interpolation when there were short periods of missing data (<300 ms). Regarding kinematic parameters calculated from Openpose data, segment and joint angles were computed using a MATLAB routine based on the estimated feature points of each joint. Each characteristic point’s positioning and definition are shown in Figure 2. The obtained marker coordinates were used to measure segment and joint angles between each characteristic point, as indicated in Table 2 [23].

Spatiotemporal gait parameters were calculated using successive heel strike and toe-off events [28]. For both legs, we measured the number of pixels between the left and right ankle key points at successive left and right heel strikes and then multiplied this value by a scaling factor to estimate step length. To estimate the scaling factor, the number of pixels between the tape marks on the walkway in the video was calculated and divided by the known distance [21].

### 2.4. Statistical Analysis

For each parameter, the assumption of normality was assessed using the Shapiro–Wilk test. However, in the statistical analysis, we applied bootstrap techniques to enhance the robustness of our results, ensuring more reliable ICC estimates. The bootstrap approach is particularly useful when data deviate from normality. Furthermore, it can improve the reliability of ICC estimates in mixed-effects models in these cases [29,30].

For the accuracy analysis, absolute errors (AEs) were computed for the kinematics parameters and for each spatiotemporal variable by calculating the absolute value after subtracting the values obtained using pose estimation methods from the value measured using marker-based motion capture, as follows [20]:

Absolute Error = abs (Motion Capture − Pose Estimation).(1)

Next, to determine whether the data obtained from Openpose agreed with the data from the optoelectronic system, intraclass correlation coefficients (ICC) (two-way mixed-effects model, absolute agreement, average measurements) were calculated for spatiotemporal and kinematic data from both Openpose and the optoelectronic system. We referred to standard interpretation criteria: ICC values between 0.5 and 0.75 indicate moderate reliability, values between 0.75 and 0.9 indicate good reliability, and values greater than 0.90 indicate excellent reliability [30].

Finally, the cross-correlation coefficients (CCCs) between both systems were used to evaluate the similarity of angles during the gait cycle [20]. The CCC values were interpreted as weak or no coupling for values between −0.3 and 0.3, moderate coupling for values between 0.3 and 0.70, or for values between −0.7 and −0.3; lastly, strong coupling for values CCC > 0.7 or CCC < −0.7 [31]. The statistical significance level was set at *p* < 0.05. SPSS 21 software (IBM, Armonk, NY, USA) was used for the statistical analysis.

The appropriate number of participants for this study was determined using GPower. To achieve a power of 80% with a 5% error level in a one-tailed test and a medium effect of 0.6, we needed 19 participants; therefore, considering a possible dropout of 5%, we enrolled 20 subjects. This sample size is reasonable and aligns with previous studies involving healthy individuals in gait analysis to compare the validity of markerless and marker-based systems. However, variability in sample sizes across different studies is notable [20,23,32,33].

## 3. Results

The mean, standard deviation (SD), mean absolute error (MAE), and the ICC of the spatiotemporal parameters for the general sample and for every group of subjects are listed in Table 3 and Table 4, respectively.

The ICC of spatiotemporal parameters in each condition was moderate to excellent in each examined case. In particular, the best reliability was found through the evaluation of the cadence; it yields good results (ICC > 0.974) both for the general sample and for each subgroup. Analyzing the MAE, particularly for gait speed in the overall sample, we found a value of 0.16, which is smaller than the MCID value proposed by Barthuly et al. [34].

For the kinematic parameters, Table 5 shows the mean, the SD, and the MAE for the Range of Motion (ROM) value of the different considered joints, and Table 6 shows the ICC (two-way mixed effects model, absolute agreement) in every group.

**Figure 3:**
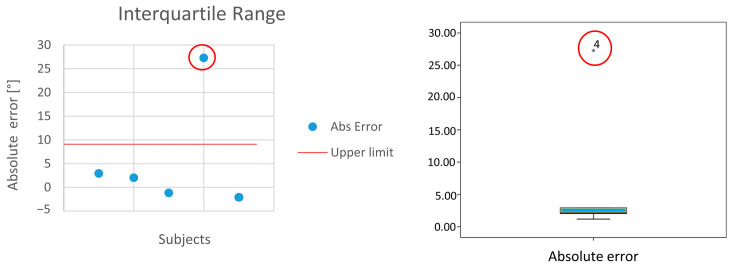
The graphs represent the interquartile range (IQR) method of outlier detection developed by John Tukey for the ROM left ankle in the healthy subjects group. The upper limit was calculated as the third quartile added in the interquartile range *1.8.

In each case, the ICC of ROM parameters was between moderate and excellent in regard to the angles on the sagittal plane. In particular, examining the general sample on the sagittal plane, all the joints showed at least good reliability, and the minimum ICC value was found in the left ankle (ICC = 0.885). Furthermore, after examining the different joints in the different subgroups, we found moderate reliability in the left ankle in the healthy subject group. Motion capture data usually include outliers [35] from skin detachment, mislabeled markers, or intentional comfort-related sources such as postural adjustments [36]. It is often useful to consider the presence of outliers that could alter the final results [37]. In this analysis, we identify the presence of possible abnormal data and evaluate the results without these values. In this case, by plotting the data as shown in Figure 3, we can identify an outlier that is affecting the analysis. Upon removal, the evaluation is excellent (ICC = 0.992 without the outlier value).

**Figure 4:**
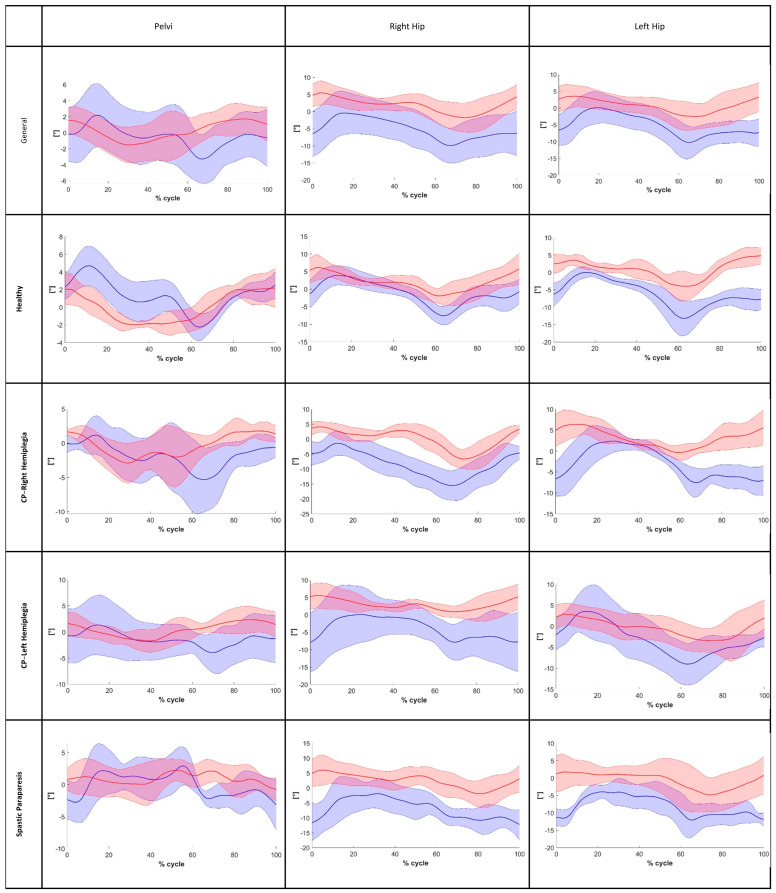
The pelvis and hip angles during the gait cycle on the frontal plane (red line: Openpose; blue line: optoelectronic system).

**Figure 5:**
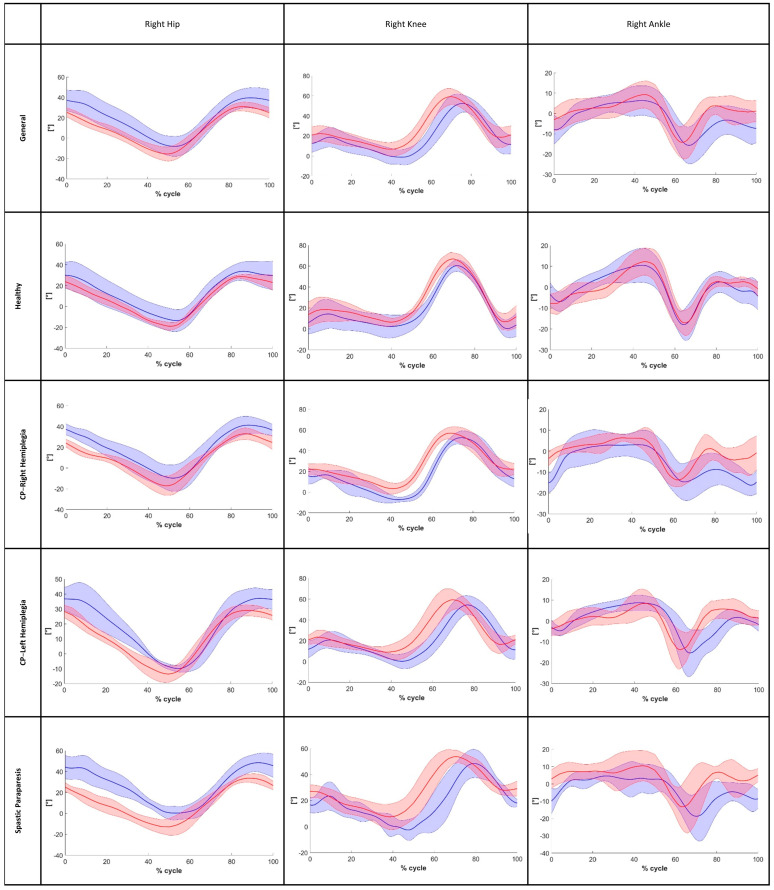
The right hip, right knee, and right ankle angles during the gait cycle on the sagittal plane (red line: Openpose; blue line: optoelectronic system).

**Figure 6:**
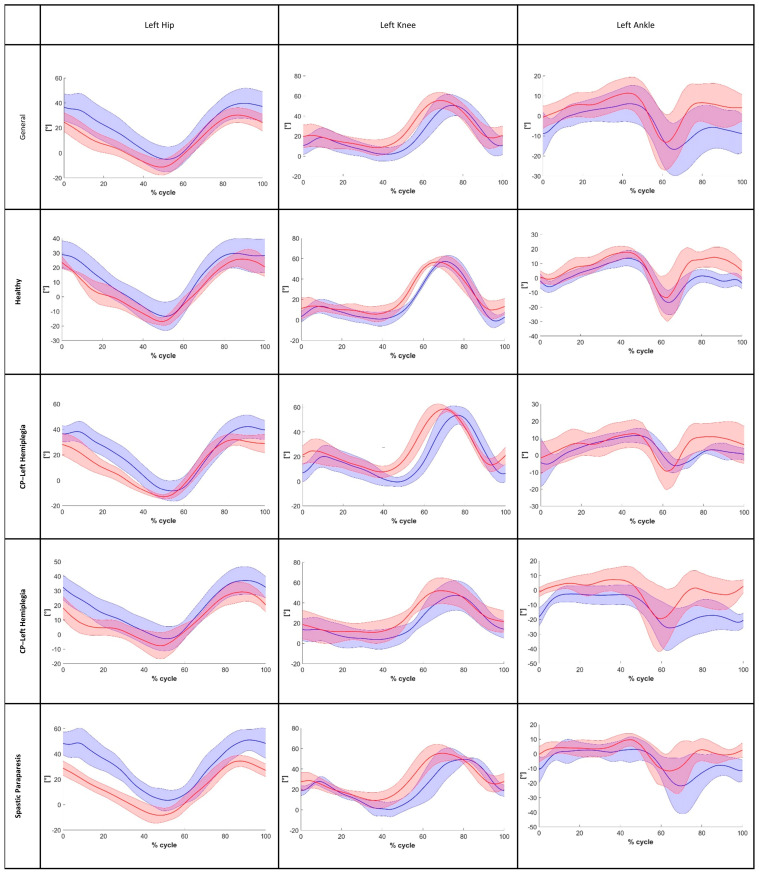
The left hip, left knee, and left ankle angles during the gait cycle on the sagittal plane (red line: Openpose; blue line: optoelectronic system).

Instead, for frontal plane angles, particularly hip abduction–adduction, poor reliability was observed. Moreover, after analyzing the MAE for the ROM on the sagittal plane in the general sample, we found values that are close to the minimal clinically important difference (MCID) reported in the literature for gait impairments relevant to our analysis. Specifically, for stroke patients’ flexion-extension movements, the literature suggests an MCID of 5.81° for the hip (in our analysis, we found a Hip-MAE = 5.41°) and 8.48° for the knee (in our analysis, we found a Knee-MAE = 5.81°) [38,39,40]. The findings for the ankle were similar to those for the hip and knee joints, with an MAE in the general sample below 4.91°. The CCC of the pelvis and the angles of the hip on the front plane (Figure 4) as well as the angles of the hip, knee, and ankle on the sagittal plane (Figure 5 and Figure 6) were calculated to assess the similarity of the angles during the walking cycle, using the values obtained by the two systems. The results are shown in Table 7.

The CCC values for joint angles in the sagittal plane ranged from moderate to strong across all examined cases, both in the overall sample and within subgroups (CCC > 0.726). Instead, for the frontal plane angles, CCC values were generally lower in both the overall sample and subgroups. In particular, for hip abduction–adduction in the spastic paraparesis group, weak coupling was observed (CCC = 0.093). The shoulder obliquity seam had a different result, namely, it showed a strong coupling in all the cases assessed (CCC > 0.791).

## 4. Discussion

This study aimed to investigate the accuracy and reliability of the Openpose method for measuring kinematic and spatiotemporal parameters of walking in the pediatric population, both with and without gait impairments. In particular, our investigation focused on whether this system could measure spatiotemporal parameters and kinematic parameters, such as joint angles, even when walking with various limitations, compared to the optoelectronic system. Overall estimates regarding spatiotemporal gait parameters were provided by Openpose with good agreement and consistency. Indeed, the analysis shows an ICC value between moderate and good reliability in all different gait patterns. In particular, the best performance with which to estimate the parameters was found in the group of healthy subjects, which showed a reliability between good and excellent (ICC > 0.769) for every metric. Moreover, the best performance to estimate the gait parameters among all groups, regarding different gait impairments, was in the cadence and gait speed, where reliability ranged from good to excellent (ICC > 0.707). This system’s ability to measure spatiotemporal parameters under various gait speed conditions with good reliability was demonstrated in previous studies conducted with a markerless pose estimation system [20,33,41]. Moreover, the MAE between the Openpose and Optoelectronic System was between 0.15 m/s and 0.18 m/s in each group; this error was less than the minimum difference with clinical relevance in patients admitted to a long-term rehabilitation facility [34].

Regarding the kinematic parameters, in particular for ROM analysis, the best reliability was obtained on the sagittal plane. In contrast, the analysis of ROM in the frontal plane was less reliable than that of the sagittal plane; in many cases, in fact, in the frontal plane, the ICC showed poor reliability. This result was in agreement with previous studies using Openpose on adult subjects [22,23]. Furthermore, the mean MAE of the ROM on the sagittal plane for the hip and knee joints was near the minimal clinically important difference and was typically less than 5°. Also, in the CCC analysis in the sagittal plane, the results were moderately strong for the hip, knee, and ankle among all groups. In particular, for the general sample and for the healthy group, the coupling was strong for all three joints. Instead, in the frontal plane, the similarity was strong only for the shoulder and was weak to moderate for the pelvis, left hip, and right hip. Moreover, the CCC values for the hip, knee, and ankle angles are similar to the results found from another study conducted with Openpose on adult subject samples [20]. These results are also similar to those obtained in a previous study on the analysis of motion without a marker with a different system compared to Openpose in the adult population [42]. This result suggests that, using a single camera, the acquisition on the sagittal plane can guarantee good reliability. Therefore, this setting could be used in clinical monitoring. Instead, a single frontal-plane video does not provide sufficient accuracy for clinical applications. Thus, considering the ICC value of the ROM, the AE, and the CCC value of the angles gait cycle, it seems that, for the kinematic parameters in the sagittal plane, Openpose estimated with high agreement and consistency. This finding supports the hypothesis that using a video camera and Openpose allows us to reliably estimate the kinematic angles of the hip, knee, and ankle during gait, as the current state of the art shows on healthy adult subjects [20,23]. Instead, the kinematic parameters on the frontal plane were estimated by the system with less performance, particularly for the ROMs, which showed some inconsistencies and low accuracy. Probably, the performance was worse on the frontal plane because the rotation angles of the hip joints and pelvis in this plane were small; therefore, the biases between the data obtained using two systems were more influenced by the motions in the transverse plane [23]. The best performance in the healthy group could be explained by the fact that we used a pretrained algorithm for the reconstruction of body key points; thus, we did not know the kind of data used for the training of the convolutional neural network, and data similar to the considered pathological group’s during training could perhaps improve the results.

This preliminary result suggests that Openpose can be used to elaborate single videos and assess kinematic parameters on the sagittal plane. Even if this application does not constitute a complete analysis of gait patterns, this method could contribute to the measuring of kinematics parameters of gait in an ecological setting, with a simple, fast, and cheap setup. This finding has potential applications in cases where traditional gait analysis is not feasible (e.g., in individuals with Autism Spectrum Disorder, where test acceptance can be challenging).

There are some limitations associated with this study, such as the small sample size of several groups to confirm this preliminary finding; future studies involving a larger number of subjects will be needed. Moreover, another limitation could be the resolution of the camera used; precedent studies have demonstrated that a better resolution of the camera can lead to better results [43]. An interesting development could be to adopt high-resolution cameras to improve the accuracy and reliability of the system. Another important limitation is that in this experiment, we used two cameras; however, each video was elaborated individually to estimate the parameters for the anatomical relative plane. Simple and fast clinical application is possible due to this simplification, avoiding the need for long and complicated calibration operations. Another future improvement could involve using two or more cameras to triangulate body point data and reconstruct 3D coordinates. Future studies on a larger population, and with more cameras with the best resolution, would be needed to verify the reliability and improve the accuracy of the system. Instead, in the case of using a single camera acquisition, future development could consider the different angles between the studied subject and the camera to assess different possible configurations as carried out by Baldinger et al. [44]. Furthermore, to assess deeply the strengths and limitations of Openpose, future studies will be required to compare it with other emerging markerless technologies.

## 5. Conclusions

This study introduces a potential setup and validates the use of Openpose for markerless kinematic gait cycle assessment in a pediatric population with various gait patterns due to different pathologies and movement impairments. Our findings confirm that the reliability observed in children is consistent with previous studies conducted on adults. In particular, this study demonstrates that by using just a low-resolution camera that is parallel to the gait direction, we can compute ROM and joint angles normalized on the gait cycle of the hip, knee, and ankle on the sagittal plane with more than good accuracy. Furthermore, this setup enables the calculation of spatiotemporal gait parameters, including cadence, gait speed, and stride length, allowing for a comprehensive characterization of the subject’s walking pattern. Despite the fact that the optoelectronic system is more accurate and reliable than a markerless system, this study demonstrates that Openpose can be a valid low-cost alternative in situations where the optoelectronic system is unaffordable for different problems (e.g., collaboration of the patients, time constraint, portability, etc.). This study was designed to compare the technical features of a markerless configuration with an optoelectronic motion analysis system. A potential future development of this setup could involve the characterization of specific gait patterns, such as foot drop, tiptoe walking, and other pathological conditions. Further studies will be necessary to validate these findings and explore their application in clinical practice.

## Figures and Tables

**Table 1:** Mean, standard deviation (SD), and range of baseline features of the sample and of different groups.

	Baseline Features (Mean—SD; Range)				
	Sample	Healthy	CP Right Hemiplegia	CP Left Hemiplegia	Spastic Paraparesis
Numbers of Subjects	20	5	5	5	5
Age [y]	11.11–SD 3.43; 5.4–17.7	9.24–SD 3.98; 5.4–15.0	11.44 –SD 3.55; 7.6–15.9	10.88–SD 3.16; 6.80–14.50	12.44–SD 3.46; 9.40–17.70
Weight [Kg]	38.64–SD 15.40; 69–17	27.40–SD 13.15; 170–49	45.50–SD 20.86; 21–69	37.40–SD 10.60; 24–49	41.60–SD 13.83; 29–59
Height [cm]	142.19–SD 21.14; 180–105	129.70–SD 24.76; 105–167.	149–SD 24.99; 113–180	142.00–SD15.03; 120–156	142.00–SD 16.63; 125–168
BMI)	18.31–SD 3.36; 27.3–13.4	15.52–SD 1.51; 13.5–17.5	19.40–SD 3.79; 15.0−24.5	18.28–SD 2.82; 13.4–20.1	20.24–SD 3.97; 17.8–27.3

**Table 2:** Definitions of segments and joints based on Openpose. Abbreviations: neck; border between cervical and thoracic vertebrae; RHip; right hip joint, LHip; left hip joint, MidHip; center of RHip and LHip, RKnee; right knee joint, RAnkle; right ankle joint, RHeel; right heel, RSmallToe; right fifth metatarsophalangeal joint.

Segments and Joints Angles	
Shoulder Elevation/Depression	The angle of a straight line connecting “RShoulder” and “LShoulder” relative to the horizontal
Pelvis Elevation/Depression	The angle of a straight line connecting “RHip” and “LHip” relative to the horizontal
Hip Flexion/Extension	The angle of a straight line connecting “RHip” and “RKnee” relative to a straight line connecting “Neck” and “MidHip
Hip Abduction/Adduction	The angle of the straight line connecting “RHip” and “RKnee” relative to the perpendicular line connecting “RHip” and “LHip”
Knee Flexion/Extension	The angle of a straight line connecting “RHip”, “RKnee” and relative to a straight line connecting “, “RKnee” and “Rankle”
Ankle Dorsiflexion/Plantar flexion	The angle of a straight line connecting “RHeel” and “RSmallToe” relative to a straight line connecting “RKnee” and “RAnkle”

**Table 3:** Mean, SD, and MAE of spatiotemporal gait parameters (OP: Openpose; OS: optoelectronic system; MAE: mean absolute error).

Metrics		OP	OS	MAE
		Mean ± SD	Mean ± SD	Mean ± SD; Max
Sample	Cadence [step/min]	56.21 ± 7.01	56.43 ± 7.08	1.63 ± 1.57; 5.9
	Gait Speed [m/s]	0.76 ± 0.16	0.92 ± 0.18	0.16 ± 0.04; 0.24
	Right Stride Length [m]	0.80 ± 0.13	0.99 ± 0.16	0.19 ± 0.04; 0.26
	Left Stride Length [m]	0.80 ± 0.15	0.98 ± 0.17	0.18 ± 0.05; 0.28
	Step Width [m]	0.13 ± 0.06	0.12 ± 0.05	0.03 ± 0.03; 0.1
Healthy	Cadence [step/min]	55.91 ± 7.20	56.80 ± 6.48	1.28 ± 2.29; 5.32
	Gait Speed [m/s]	0.83 ± 0.13	0.97 ± 0.13	0.16 ± 0.24; 0.18
	Right Stride Length [m]	0.89 ± 0.14	1.04 ± 0.16	0.15 ± 0.04; 0.19
	Left Stride Length [m]	0.88 ± 0.16	1.05 ± 0.17	0.18 ± 0.04; 0.23
	Step Width [m]	0.09 ± 0.04	0.10 ± 0.04	0.01 ± 0.01; 0.03
CP Right Hemiplegia	Cadence [step/min]	57.13 ± 7.10	56.04 ± 8.94	1.96 ± 0.81; 2.62
	Gait Speed [m/s]	0.80 ± 0.12	0.96 ± 0.14	0.16 ± 0.03; 0.19
	Right Stride Length [m]	0.83 ± 0.09	1.03 ± 0.12	0.21 ± 0.04; 0.25
	Left Stride Length [m]	0.83 ± 0.11	1.03 ± 0.13	0.20 ± 0.05; 0.28
	Step Width [m]	0.13 ± 0.03	0.12 ± 0.01	0.02 ± 0.01; 0.04
CP Left Hemiplegia	Cadence [step/min]	56.54 ± 7.18	58.80 ± 7.82	2.35 ± 2.28; 5.90
	Gait Speed [m/s]	0.74 ± 0.22	0.92 ± 0.26	0.18 ± 0.04; 0.22
	Right Stride Length [m]	0.75 ± 0.16	0.95 ± 0.21	0.20 ± 0.06; 0.26
	Left Stride Length [m]	0.76 ± 0.19	0.94 ± 0.20	0.17 ± 0.06; 0.24
	Step Width [m]	0.14 ± 0.08	0.13 ± 0.07	0.03 ± 0.03; 0.09
Spastic Paraparesis	Cadence [step/min]	55.26 ± 8.79	55.08 ± 6.45	1.91 ± 1.59; 3.98
	Gait Speed [m/s]	0.70 ± 0.18	0.85 ± 0.21	0.15 ± 0.04; 0.19
	Right Stride Length [m]	0.12 ± 0.05	0.14 ± 0.06	0.03 ± 0.02; 0.21
	Left Stride Length [m]	0.73 ± 0.12	0.91 ± 0.18	0.18 ± 0.06; 0.23
	Step Width [m]	0.12 ± 0.05	0.14 ± 0.06	0.03 ± 0.02; 0.05

**Table 4:** ICC values for the gait parameters and accuracy levels (+++ = excellent, ++ = good, + = moderate) [30].

	Sample		Healthy		CP—Right Hemiplegia		CP—Left Hemiplegia		Spastic Paraparesis	
	ICC	ICC level	ICC	ICC level	ICC	ICC level	ICC	ICC level	ICC	ICC level
Cadence [step/min]	0.974	+++	0.999	+++	0.983	+++	0.987	+++	0.975	+++
Gait Speed [m/s]	0.811	++	0.784	++	0.707	+	0.868	++	0.859	++
Right Stride Length [m]	0.687	+	0.781	++	0.514	+	0.753	++	0.658	+
Left Stride Length [m]	0.734	+	0.769	++	0.566	+	0.813	++	0.713	+
Step Width [m]	0.846	++	0.936	+++	0.500	+	0.909	+++	0.893	++

**Table 5:** Mean, SD, MAE for articular ROM (OP: Openpose; OS: optoelectronic system, MAE: mean absolute error).

Metrics		OP	OS	MAE
		Mean ± SD	Mean ± SD	Mean ± SD; MAX
Sample	ROM_ShoulderObliquity [°]	5.99 ± 4.43	7.02 ± 4.25	1.59 ± 2.02; 8.60
	ROM_PelviObliquity [°]	6.40 ± 1.83	8.08 ± 3.11	2.34 ± 1.87; 8.30
	ROM r_hip_AA [°]	9.50 ± 3.36	13.53 ± 3.92	4.70 ± 3.71; 12.66
	ROM l_hip_AA [°]	9.18 ± 3.13	13.74 ± 3.47	4.56 ± 2.53; 10.08
	ROM r_hip_FE [°]	47.89 ± 7.79	50.38 ± 7.40	3.19 ± 2.85; 11.68
	ROM l_hip_FE [°]	43.39 ± 7.61	47.64 ± 8.78	5.41 ± 3.30; 10.61
	ROM r_knee_FE [°]	55.28 ± 9.66	59.23 ± 8.94	5.81 ± 2.47; 11.89
	ROM l_knee_FE [°]	50.09 ± 11.19	54.27 ± 11.54	4.98 ± 2.81; 12.18
	ROM r_ankle_FE [°]	27.05 ± 7.97	27.84 ± 8.67	3.61 ± 2.55; 9.23
	ROM l_ankle_FE [°]	29.90 ± 14.20	29.01 ± 13.85	4.97 ± 7.67; 27.29
Healthy	ROM_ShoulderObliquity [°]	3.06 ± 1.10	3.36 ± 1.79	0.77 ± 0.45; 1.20
	ROM_PelviObliquity [°]	5.98 ± 1.26	7.10 ± 2.81	2.12 ± 1.32; 4.27
	ROM r_hip_AA [°]	10.19 ± 3.29	11.64 ± 1.60	2.58 ± 0.67; 3.11
	ROM l_hip_AA [°]	9.88 ± 4.17	14.26 ± 5.34	4.38 ± 2.48; 7.82
	ROM r_hip_FE [°]	48.36 ± 4.81	48.54 ± 4.04	1.62 ± 1.03;2.93
	ROM l_hip_FE [°]	45.32 ± 6.30	45.24 ± 3.93	3.99 ± 2.98; 8.08
	ROM r_knee_FE [°]	63.03 ± 3.39	62.34 ± 6.16	4.95 ± 1.03; 6.28
	ROM l_knee_FE [°]	55.27 ± 8.52	60.42 ± 5.39	5.77 ± 2.82; 8.07
	ROM r_ankle_FE [°]	31.20 ± 8.33	29.92 ± 9.98	3.23 ± 2.03; 5.92
	ROM l_ankle_FE [°]	38.50 ± 12.40	32.70 ± 12.13	7.11 ± 11.30; 27.29
CP—Right Hemiplegia Healthy	ROM_ShoulderObliquity [°]	4.90 ± 4.91	7.22 ± 3.65	2.32 ± 3.60; 8.60
	ROM_PelviObliquity [°]	7.17 ± 2.78	8.84 ± 2.07	1.74 ± 1.21; 3.26
	ROM r_hip_AA [°]	11.93 ± 2.45	15.34 ± 4.59	4.29 ± 3.54; 10.19
	ROM l_hip_AA [°]	8.77 ± 3.15	13.78 ± 1.29	5.01 ± 2.08; 7.78
	ROM r_hip_FE [°]	50.34 ± 10.42	51.74 ± 8.06	2.74 ± 2.39; 4.98
	ROM l_hip_FE [°]	46.51 ± 3.97	51.62 ± 4.62	5.11 ± 3.56; 9.95
	ROM r_knee_FE [°]	55.33 ± 10.45	62.40 ± 8.33	7.07 ± 3.26; 11.89
	ROM l_knee_FE [°]	53.04 ± 3.17	56.30 ± 4.11	3.72 ± 2.28; 6.78
	ROM r_ankle_FE [°]	23.38 ± 2.83	24.76 ± 7.16	3.77 ± 3.66; 9.23
	ROM l_ankle_FE [°]	23.59 ± 12.49	23.82 ± 11.32	1.40 ± 1.19; 3.17
CP—Left Hemiplegia Healthy	ROM_ShoulderObliquity [°]	4.92 ± 1.76	6.04 ± 1.69	1.12 ± 0.40; 1.59
	ROM_PelviObliquity [°]	5.93 ± 1.57	6.82 ± 2.48	2.10 ± 1.22; 3.51
	ROM r_hip_AA [°]	6.24 ± 3.11	13.46 ± 3.17	7.22 ± 3.19; 12.25
	ROM l_hip_AA [°]	9.47 ± 3.11	14.64 ± 4.15	5.17 ± 3.73; 10.08
	ROM r_hip_FE [°]	44.88 ± 5.40	49.32 ± 8.26	4.44 ± 4.19; 11.68
	ROM l_hip_FE [°]	37.88 ± 10.32	42.06 ± 13.18	4.78 ± 4.31; 10.61
	ROM r_knee_FE [°]	54.70 ± 7.41	57.32 ± 9.24	4.43 ± 2.61; 7.91
	ROM l_knee_FE [°]	43.93 ± 17.12	46.54 ± 19.28	4.73 ± 2.34; 7.69
	ROM r_ankle_FE [°]	26.66 ± 10.78	28.92 ± 9.06	4.56 ± 2.53; 7.31
	ROM l_ankle_FE [°]	29.42 ± 17.97	27.92 ± 13.06	4.58 ± 3.68; 10.50
Spastic Paraparesis Healthy	ROM_ShoulderObliquity [°]	11.07 ± 4.34	11.46 ± 4.89	2.14 ± 1.99; 4.80
	ROM_PelviObliquity [°]	6.53 ± 1.69	9.56 ± 4.57	3.40 ± 3.14; 8.30
	ROM r_hip_AA [°]	9.64 ± 2.44	13.66 ± 5.56	4.70 ± 5.32; 12.66
	ROM l_hip_AA [°]	8.58 ± 2.87	12.26 ± 2.40	3.68 ± 2.09; 5.74
	ROM r_hip_FE [°]	47.97 ± 10.41	51.92 ± 9.97	3.95 ± 2.87; 7.83
	ROM l_hip_FE [°]	43.87 ± 7.64	51.62 ± 8.36	7.75 ± 1.25; 8.91
	ROM r_knee_FE [°]	48.08 ± 11.29	54.86 ± 11.55	6.78 ± 2.03; 8.52
	ROM l_knee_FE [°]	48.10 ± 11.07	53.82 ± 9.35	5.72 ± 3.93; 12.18
	ROM r_ankle_FE [°]	26.94 ± 8.32	27.74 ± 10.28	2.88 ± 2.24; 5.74
	ROM l_ankle_FE [°]	28.08 ± 13.35	31.60 ± 20.12	6.78 ± 10.53; 25.32

**Table 6:** ICC values for articular ROM and accuracy levels (+++ = excellent, ++ = good, + = moderate, − = poor) [30].

	Sample		Healthy		CP—Right Hemiplegia		CP—Left Hemiplegia		Spastic Paraparesis	
	ICC	ICC Level	ICC	ICC Level	ICC	ICC Level	ICC	ICC Level	ICC	ICC Level
ROM Shoulder Obliquity [°]	0.909	+++	0.904	+++	0.757	++	0.893	++	0.896	++
ROM Pelvis Obliquity [°]	0.605	+	0.557	+	0.830	++	0.482	−	0.530	+
ROM right hip AA [°]	0.280	−	0.674	+	0.298	−	0.241	−	0.510	+
ROM left hip AA [°]	0.535	+	0.767	++	0.338	−	0.407	−	0.532	+
ROM right hip FE [°]	0.920	+++	0.953	+++	0.964	+++	0.852	++	0.947	+++
ROM left hip FE [°]	0.850	++	0.696	+	0.574	+	0.931	+++	0.805	++
ROM right knee FE [°]	0.882	++	0.591	+	0.851	++	0.904	+++	0.912	+++
ROM left knee FE [°]	0.938	+++	0.812	++	0.665	+	0.980	+++	0.896	++
ROM right ankle FE [°]	0.925	+++	0.957	+++	0.709	+	0.930	+++	0.963	+++
ROM left ankle FE [°]	0.885	++	0.666	+	0.995	+++	0.966	+++	0.865	++

**Table 7:** CCC values joint angles in every group and their levels (+++ = strong, ++ = moderate, + = weak) [31].

	Sample		Healthy		CP—Right Hemiplegia		CP—Left Hemiplegia		Spastic Paraparesis	
	CCC	CCC Level	CCC	CCC Level	CCC	CCC Level	CCC	CCC Level	CCC	CCC Level
Shoulder obliquity [°]	0.971	+++	0.791	+++	0.921	+++	0.859	+++	0.979	+++
Pelvic obliquity [°]	0.187	+	0.389	+	0.221	+	0.194	+	0.093	+
Right hip AA [°]	0.713	+++	0.749	+++	0.748	+++	0.116	+	0.543	++
Left hip AA [°]	0.667	++	0.611	++	0.084	+	0.836	+++	0.627	++
Right hip FE [°]	0.958	+++	0.993	+++	0.977	+++	0.930	+++	0.887	+++
Left hip FE [°]	0.957	+++	0.982	+++	0.924	+++	0.956	+++	0.930	+++
Right knee FE [°]	0.887	+++	0.979	+++	0.924	+++	0.773	+++	0.761	+++
Left knee FE [°]	0.896	+++	0.956	+++	0.801	+++	0.934	+++	0.825	+++
Right ankle FE [°]	0.813	+++	0.933	+++	0.776	+++	0.633	++	0.656	++
Left ankle FE [°]	0.726	+++	0.854	+++	0.627	++	0.713	+++	0.745	+++

## Data Availability

Data presented in this study are available on request (and protocol approved by EC) to the corresponding author in anonymous form due to GDPR and informed consent.
